# Analysis of the Sugar Content in Food Products by Using Gas Chromatography Mass Spectrometry and Enzymatic Methods

**DOI:** 10.3390/foods7110185

**Published:** 2018-11-08

**Authors:** Najah M. Al-Mhanna, Holger Huebner, Rainer Buchholz

**Affiliations:** 1 Engineering Department, Faculty of Engineering and Computer Science, German University of Technology in Oman, P.O. Box 1816, Athaibah PC 130, Oman; 2 Institute of Bioprocess Engineering, Friedrich-Alexander-University Erlangen-Nuremberg, Paul-Gordan-Str. 3, 91052 Erlangen, Germany; holger.huebner@fau.de (H.H.); rainer.buchholz@bvt.cbi.uni-erlangen.de (R.B.)

**Keywords:** gas chromatography mass spectrometry, sugar content in food, date juice (syrup), sugar quantification and qualification, enzymatic methods

## Abstract

The aim of this study is to develop and optimise a method of sugar content determination in food products. Date juice (syrup) was used as a sample natural food resource for the analysis because of its potential usage as an alternative substrate for a variety of fermentation processes. Hence, qualifying and quantifying its sugar content is a crucial step. Therefore, gas chromatography mass spectrometry (GCMS) was used as a pre-qualitative method to identify the types of sugar in the date sample. The results demonstrate that the analysed date juice contains glucose, fructose and sucrose. This analysis was obtained by measuring the retention time of individual standard sugar samples such as glucose, fructose, mannose and sucrose. In addition, the mass spectra of the standard and date juice samples contained characteristic fragments of glucose, fructose and sucrose. Thus, GCMS results determined the appropriate enzymatic assays for quantifying the sugars in date juice. These results were similar to those of the two enzymatic methods (standard enzymatic assay and measuring the change in pH by CL10 analyser). Therefore, they confirmed the identified sugars and provided the sugar contents of the sample. Consequently, sugar quantification results indicate that 1 g of date juice sample contains a total of 0.5275–0.5507 g of six-carbon sugars (glucose + fructose) and 0.064–0.068 g of sucrose. As a consequence, the total sugar content in 1 g of date juice is 0.600–0.615 g. These results are comparable to the sample analysis that is provided by the date juice production company.

## 1. Introduction

Date juice (syrup) is the most common product of date palm (Phoenix dactylifera). Date juice is one of the richest foodstuffs in neutral compounds, such as monosaccharides, disaccharides, mineral salts and vitamins. In addition, its protein content (1.5–3% *w*/*w*) is low. These substances are essential for the growth of microorganisms, especially yeast. Date extracts are used in different industrial processes. Utilization of poor-quality dates and date by-products has been studied in baked goods and ice cream, to produce caramel colour, alcohol, vinegar, citric acid and oxytetracycline and as a thermophilic dairy starter [1,2]. Moreover, date juice can be used as an alternative substrate for single-cell protein production [2]. Other examples of using date juice as a substrate in fermentations can be found in Refs. [3,4,5,6].

Therefore, the contents of date juice, particularly its sugars (which will serve as a carbon source for many fermentation processes) must be analysed in more detail. Although many reports have defined date sugar contents, for example, mono- and disaccharide sugars, such as glucose, fructose, mannose and sucrose, this determination is limited by the source of date palm. As a consequence, the sugar contents of date juices vary considerably according to the source of the palm fruits. There are at least 2000 different cultivars of date palm; therefore, date sugar contents are accordingly diverse. In addition, the sucrose content varies during the different growth states of the date fruit. For instance, sucrose increases rapidly as the fruits mature from the kimri stage to the khalal stage, while it decreases to a non-detectable level at the tamr stage, that is, sucrose is completely converted to fructose and glucose [1,5,6,7,8].

Gas chromatography mass spectrometry (GCMS) is widely used for sugar identification and quantification. Consequently, the reduced sugars must be oximated before analysis to block the prochiral moiety of the reduced sugar molecules. As a result, two new structural isomers, the syn and anti-forms, are obtained. Otherwise, five forms for each reduced sugar will be formed during the GCMS process. Oximation is not conducted for non-reduced sugars, for example, disaccharides, because they are devoid of prochiral centres [9,10,11]. To derivatize a functional group, such as (-OH), a silylation reagent, for example, trimethylsilyl (TMS) must be used. The produced derivatives are less polar and more volatile. Silylation must be applied for both reduced and non-reduced sugars [12,13].

### Research Aim and Outcome

Different methods are currently used to quantify sugars. Enzymatic methods are the most common quantification methods for defined sugars. These methods are based on several enzymatic reactions, such as sucrose hydrolysis and phosphorylation of glucose and fructose. The assays are based on measuring either the change in pH of the sample using a pH sensor device [14] or by measuring the increase in the absorbance value according to the standard assay of a sugar kit [15]. Both methods depend on the sequences of the enzymatic reactions. However, such analysis is difficult and costly for evaluating the unknown sugar content of a natural product, for example, date juice, which has varying sugar contents. There are tremendous sources of dates; therefore, date sugar contents are also diverse. In addition, the sucrose content decreases with the ripening of the date fruit and can be converted completely to fructose and glucose during the ripening process. Therefore, in this study, the intention is to identify the sugar contents in a date juice sample using GCMS as a pre-qualitative method. Consequently, this study will determine the proper enzymatic methods for quantifying the sugars in date juice. The research novelty is in combination of three different methods to determine the sugar content of food product. Furthermore, it introduces a new method of using CL10 device (mpH change measuring analyser device) in quantification of date sugar content. Additionally, no similar studies are available.

Date juice was selected as the sample of analysis because of its potential use in bioproduction and food products. Moreover, date juice matrix content (e.g., glucose, fructose, sucrose, minerals (i.e., rich sources of selenium, copper, phosphorus, potassium and magnesium), protein, phenolics, fat, a moderate source of vitamins (e.g., B6, B9, B2 and B3), low concentrations of vitamins B1, C and A) is similar to the composition of the date fruit as can be found in the literature [16,17,18]. Furthermore, measuring the sugar content in a date fruit requires sample preparation. The preparation step should be able to extract the sugar content completely. Pressing date fruit, to recover sugars, is not a feasible commercial process and it leads to have impurities as a consequence of breaking down the hemicellulose wall. In addition, only a fraction of the sugar content can be recovered [19]. One of the common and economical method of sugar extraction is by heating the date fruit with water at a temperature below 70 °C, since the solubility proportional directly with temperature. However, high temperature (e.g., above 70 °C) may change the chemical composition of the extracted solution. Moreover, such extraction process is affected by the time, mixing, date flesh- water contact area and water temperature [20]. This extraction process is similar to date juice production process. Therefore, date juice represents a final product, of date fruit processing, that contains the extracted soluble sugars of a date fruit. Consequently, the current study can be used to analysis the sugar content of date fruit.

Date juice matrix contains elements that are almost similar to some other fruits’ juices or food products. For instance, 100 g honey contains water 15–20 g, fructose 30–45 g, glucose 24–40 g, sucrose 0.1–4.8 g, trisaccharides (melezitose 0.3–22.0, erlose 0.5–6 g), minerals 0.6–2.0 g and proteins 0.2–0.7 g [21]. Moreover, the sample size, that was used in this study, was very small (10–21.5 µL) in comparison to the total solution sample (521–775 µL) as described in Section 2, material and methods. This makes the measurement free of noise and it eliminates the effect of other constituents beside the sugars. Therefore, the study shows a potential extension in determination the sugar content of other food products.

In this research, preparation of low sugar concentration calibration curves were intended. The reason of developing low concentration measurement assay is to provide an assay that can be used to measure wide range of sugar concentration, since high sugar concentration samples can be diluted before the measurement. Al-Mhanna et al. [14] optimized the operating conditions of the pH-change analyser device to be utilized in measuring low glucose concentration. Moreover, the absorbance measurements deviate from linearity for absorbance values above 1. Therefore, spectrophotometers absorbance range is between 0.1–1 (linear) because the absorbance is calculated from the log (Light intensity through blank/light intensity through a sample). Therefore, diluting the sample is necessary.

## 2. Materials and Methods

The date juice (syrup) sample was produced by Monalisa Trade AB (Sweden). The composition of the date juice was provided by the company. Sugars, such as d-glucose, fructose, mannose and sucrose, were bought from Carl Roth GmbH. N,O-Bis(trimethylsilyl)trifluoroacetamide (BSTFA) and TMS were purchased from Sigma-Aldrich Co. Various methods were implanted in the experimental work that described below

### 2.1. Sugar Identification Using GCMS

#### 2.1.1. Standard Sugar Preparation

Standard sugar solutions were prepared separately by dissolving 10 mg of glucose, fructose, mannose, or sucrose in 800 µL of pyridine. For GCMS analysis, these solutions were subjected to the oximation-silylation step, which is described later.

#### 2.1.2. Date Juice Sample Preparation

A small amount of date syrup (20 mg) was dried (55 °C, 10 mbar, 2 h) using a rotational vacuum concentrator (Christ Rvc 2-33IR). The dried substance was dissolved in 1000 µL of pyridine and placed in an ultrasonic bath for 5 min. In addition, the sample was mixed well by vortex for 20 s. The sample was centrifuged (12,000× *g*, 10 min, 20 °C) to remove any insoluble materials. Part of the supernatant, 800 µL, was taken for the oximation-silylation step, which is described later.

#### 2.1.3. Oximation and Silylation

Hexose sugars, such as glucose, fructose and mannose, in standard solutions or sugars in date juice samples were oximated prior to GCMS analysis. The oximation was achieved by adding 50 mg of O-methyl hydroxylamine-hydrochloride to 800 µL of the standard sugar solution or date juice solution. The solutions were incubated at 95 °C for 45 min on a heat plate.

Then, 200 µL of the silylation reagent, BSTFA, was added to each oximated sample. The mixture was incubated at 90 °C for 30 min on a heat plate. Then, the samples were centrifuged (8000× *g*, 10 min, 20 °C). The standard sucrose solution was silylated following the same procedure. However, the incubation time was extended to 60 min. Finally, the derivatized samples were transferred to the GCMS device for analysis.

#### 2.1.4. Measurements

The derivatized sample (1 µL) was automatically injected into the GCMS and the measurement began immediately. The GCMS parameters were optimized and are described in Table 1.

### 2.2. Sugar Quantification

To analyse the sugar contents, the following two methods were used.

#### 2.2.1. Sugar Quantification Using a Delta pH Device (First Method)

##### Procedure

The differential pH device was set at a constant temperature of 37 °C, waiting time of 4 s and maximum reaction time (cycle time) of 200 s. The first run was performed for the buffer only to determine the noise of the device. Then, 10 µL of the sample was manually added to the mixing chamber of the device using a micropipette. After 40 s, the measurement cycle was started and about 315 µL of this solution was then automatically distributed into the tubes leading to electrodes 1 and 2. To add a certain amount of enzyme to the mixing chamber, which included about 775 µL of remaining solution, 10 µL of the hexokinase enzyme (1 U/5 µL) was injected using a micropipette. Different pH values were observed at electrode 1 and electrode 2 for all samples. The time required to achieve these reactions was different for each sample and depended on the glucose concentration (time is proportional to 1/[glucose]). This procedure was performed to obtain a calibration curve from different glucose concentrations.

##### Enzyme Preparation

After preparing 1.5 mL of solutions containing 25 vol% glycerol in bidest water, enzyme was added to 1 mL of this solution. The mixture was mixed using a vortex device.

##### Date Juice Sugar Determination

Date juice was quantified by measuring the change in pH for sucrose, an additional hydrolysis step was performed to convert sucrose to fructose and glucose. The glucose concentration was measured before and after hydrolysis to determine the sucrose concentration as follows:

[Sucrose] = [glucose] Total (after hydrolysis) − [glucose] Initial (before hydrolysis)(1)

The hydrolysis step was performed by adding 10 µL of the invertase enzyme (3.2 U/µL) to 1 mL of date juice at pH 4.7. The mixture was mixed well in a thermo-mixer at 37 °C and 350 rpm for 30 min.

#### 2.2.2. Sugar Quantification Using a Standard Enzymatic Assay (Second Method)

##### Materials

Six solutions were purchased from Megazyme and were prepared for sugar quantification as follows:

1. Solution 1: Imadazol buffer (2 M, pH 7.6) + MgCl2 (100 mM) + sodium azide (0.02% *w*/*v*)
2. Solution 2: NADP+ (12.5 mg/mL) + ATP (36.7 mg/mL)
3. Suspension 3: Hexokinase (425 U/mL) + glucose-6-phosphate dehydrogenase (212 U/mL)
4. Suspension 4: Phosphoglucose isomerase (1000 U/mL)
5. Solution 5: Sugar solutions that were obtained by using bidest water 0.005 µS as described later.
6. Solution 6: β-Fructosidase (200 U/mL) dissolved in citrate buffer (pH 4.6)

##### Preparation of a Glucose Calibration Curve

First, a calibration curve was prepared. Samples of different glucose concentrations (0–1 g/L) were prepared by dissolving glucose in bidest water. The blank contained 0 g/L of glucose. The absorbance value of the blank sample was subtracted from all measured values.

A total volume of 521 µL was prepared for each sample, as shown in Table 2. The samples were mixed and incubated at 30 °C for 3 min. Then, the absorbance of the samples was measured at 340 nm. A calibration curve was prepared by plotting the obtained absorbance values against glucose concentrations, as shown in the results.

##### Determination of the Sugars in the Date Juice (Syrup) Sample

Because date juice (syrup) contains sucrose, fructose and glucose, a hydrolysis step was performed. The glucose and fructose concentrations were calculated before hydrolysis. The total glucose content was measured after the hydrolysis step. Thus, the sucrose concentration was calculated by subtracting the initial glucose and fructose contents from the total glucose measured after hydrolysis. This glucose calculation was performed by comparing the obtained absorbance values with the glucose calibration curve.

##### Before Hydrolysis

*Glucose Determination*

The initial glucose content in date juice was determined. The absorbance at 340 nm was measured according to the procedure outlined for preparing the glucose calibration curve.

*Fructose Determination*

The initial sugar (glucose + fructose) was measured by preparing 521-µL mixed samples and the total volume of the solutions is described in Table 2. The samples were incubated at 30 °C for 5 min.

The absorbance of this solution was measured at 340 nm. By comparing the value of absorbance with the glucose calibration curve, the total sugar (glucose + fructose) was determined. The fructose concentration was obtained by subtracting the initial glucose concentration from the total sugar measurement.

##### After Hydrolysis

*Sucrose Determination*

The sample (21.5 µL) was mixed with 43 µL of solution 6. Then, the mixture was incubated at 37 °C for 10 min. Thus, a sample of 521 µL of different solutions was prepared and mixed well, as outlined in Table 2.

After 10 min of incubation at 30 °C, the absorbance was measured at 340 nm. The absorbance value demonstrated the entire sugar content in terms of total glucose. Subtracting the calculated initial sugar content (glucose + fructose) from this total sugar content provided the sucrose concentration.

## 3. Results

### 3.1. Qualitative Analysis Using Gas Chromatography Mass Spectrometry

**Figure 1:**
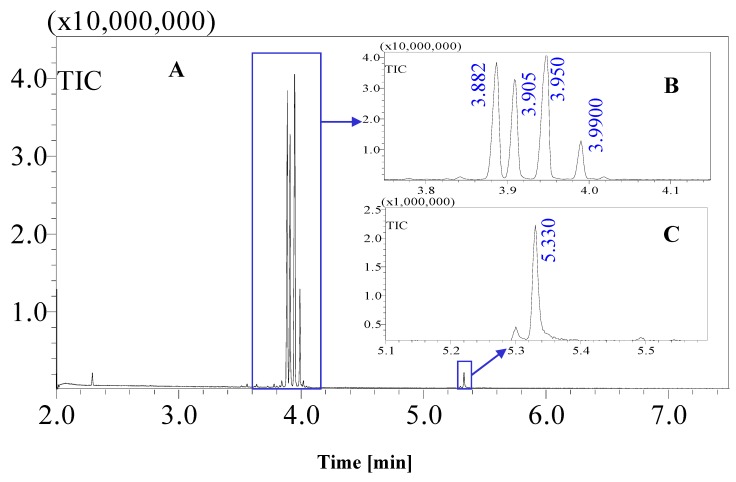
Total ion chromatogram (TIC) of an oximated-silylated date juice sample. Several peaks were obtained at different retention times (e.g., 2.3, 3.88, 3.905, 3.95, 3.99, 5.3 and 5.33 min). (**A**) Whole date juice chromatogram for a 7-min measurement; (**B**) the date juice chromatogram from 3.8–4.1 min; (**C**) the date juice chromatogram from 5.1–5.5 min.

**Figure 2:**
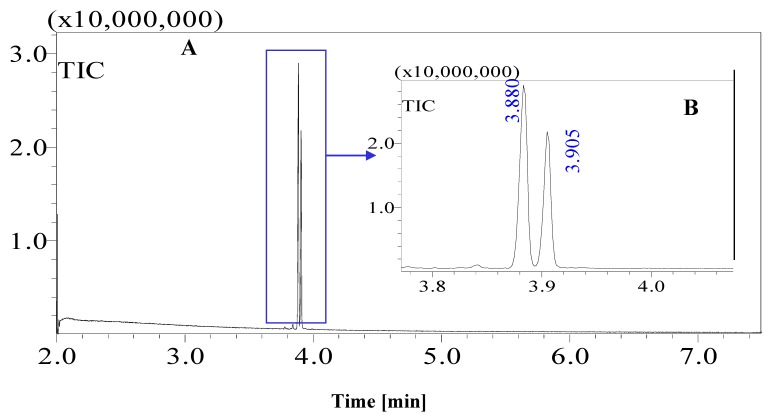
Total ion chromatogram (TIC) of standard fructose. A fructose sample was oximated and silylated. Syn and anti-forms of fructose were observed at 3.88 and 3.905 min, respectively. (**A**) Whole chromatogram of standard fructose for a 7-min measurement; (**B**) the fructose chromatogram from 3.8–4.0 min.

**Figure 3:**
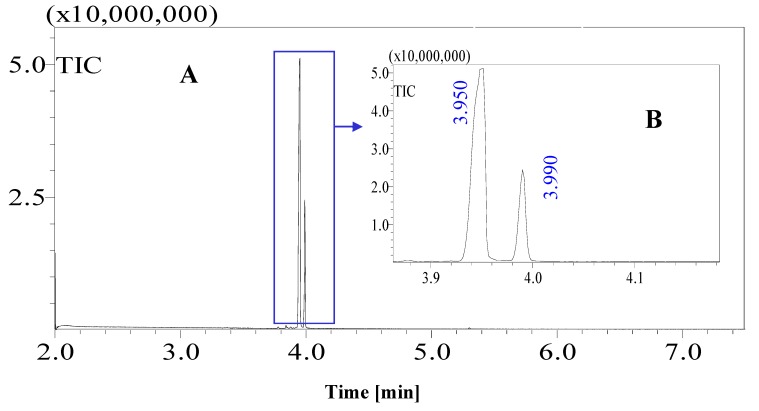
Total ion chromatogram (TIC) of standard d-glucose. Chromatograph of the oximated and silylated glucose sample was obtained. Glucose syn and anti-forms were observed at 3.95 and 3.99 min. (**A**) Total chromatogram of a standard glucose sample for a 7-min measurement; (**B**) the glucose standard sample from 3.9–4.1 min.

**Figure 4:**
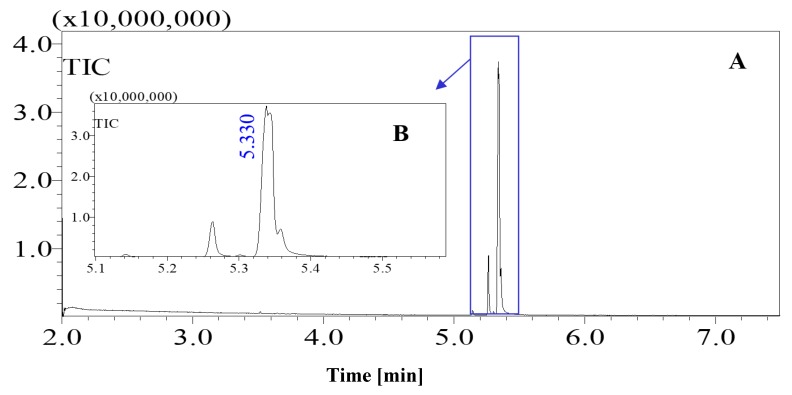
Total ion chromatogram (TIC) of standard sucrose. A chromatograph of a silylated sucrose sample. (**A**) Whole chromatogram of standard sucrose for a 7-min measurement; (**B**) chromatogram of standard sucrose from 5.1–5.5 min.

**Figure 5:**
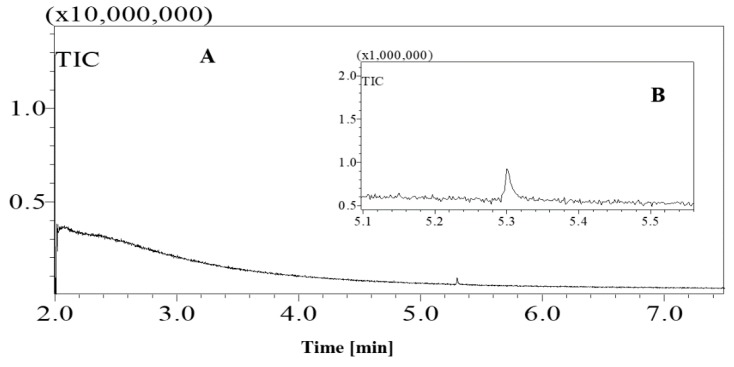
Total ion chromatogram (TIC) of the blank sample (pyridine). (**A**) Whole chromatogram of the blank sample for a 7-min measurement; (**B**) chromatogram of the blank sample from 5.1–5.5 min.

Standard sugars, such as d-glucose, fructose and sucrose and date juice samples were analysed under the same GCMS operating conditions (see Table 1). The measurements were performed under optimal operating conditions; other operating conditions gave either no results or noise. Moreover, sharp and distinguished peaks were obtained, as shown in Figure 1, Figure 2, Figure 3 and Figure 4. Figure 1 shows the chromatogram of a date juice sample. The results reveal main peaks at retention times of 3.88, 3.905, 3.95, 3.90 and 5.33 min. These peaks correspond to the peaks obtained for standard sugars, that is, fructose (Figure 2), d-glucose (Figure 3) and sucrose (Figure 4). Additionally, the chromatogram of the blank pyridine sample contains a small peak at a retention time of 5.3 min, as demonstrated in Figure 5.

**Figure 6:**
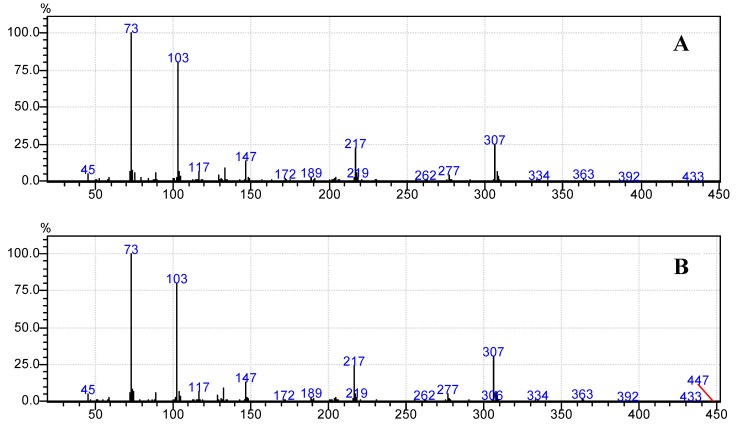
Mass spectrum at a retention time of 3.88 min. (**A**) Standard fructose; (**B**) Date juice.

**Figure 7:**
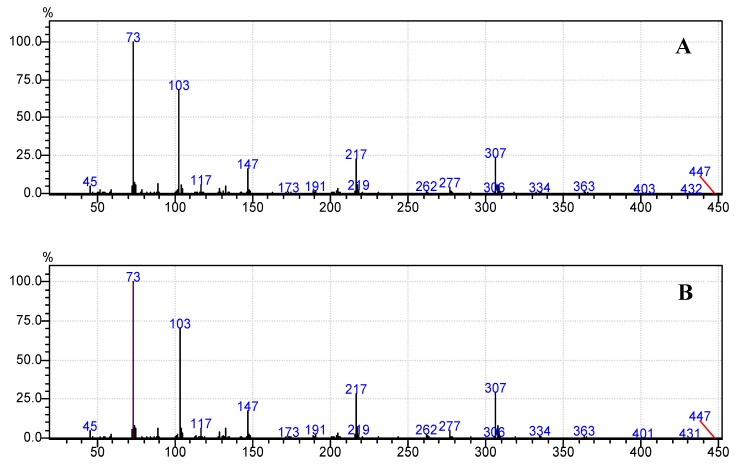
Mass spectrum at a retention time of 3.905 min. (**A**) Standard fructose; (**B**) Date juice.

**Figure 8:**
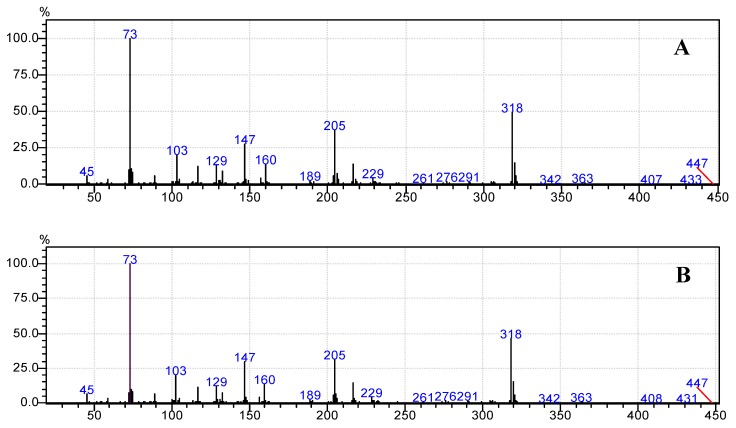
Mass spectrum at a retention time of 3.95 min. (**A**) Standard d-glucose; (**B**) Date juice.

**Figure 9:**
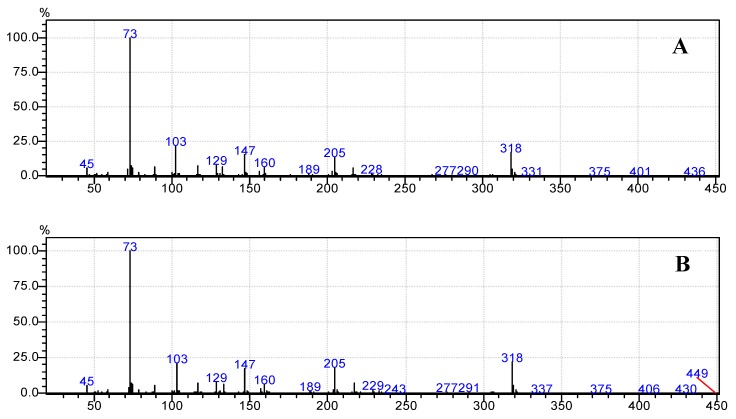
Mass spectrum at a retention time of 3.99 min. (**A**) Standard d-glucose; (**B**) Date juice.

**Figure 10:**
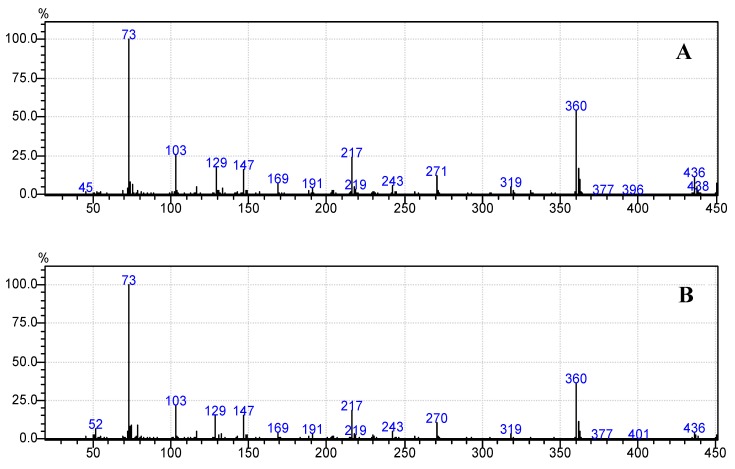
Mass spectrum of the date juice sample at a retention time of 5.33 min. (**A**) Standard sucrose; (**B**) Date juice.

**Figure 11:**
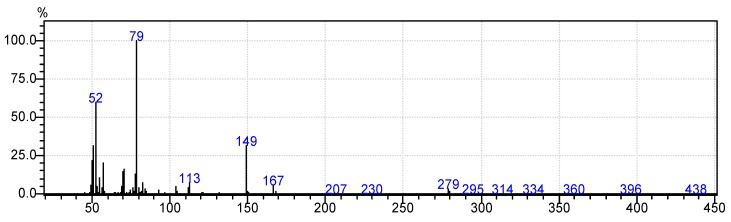
Mass spectrum of a blank sample (pyridine) at a retention time of 5.3 min.

Moreover, Figure 6b, Figure 7b, Figure 8b, Figure 9b and Figure 10b demonstrate that the spectra of date juice match the spectra of standard sugars, as shown in Figure 6a, Figure 7a, Figure 8a, Figure 9a and Figure 10a. Additionally, the spectrum of pyridine is presented in Figure 11.

**Figure 12:**
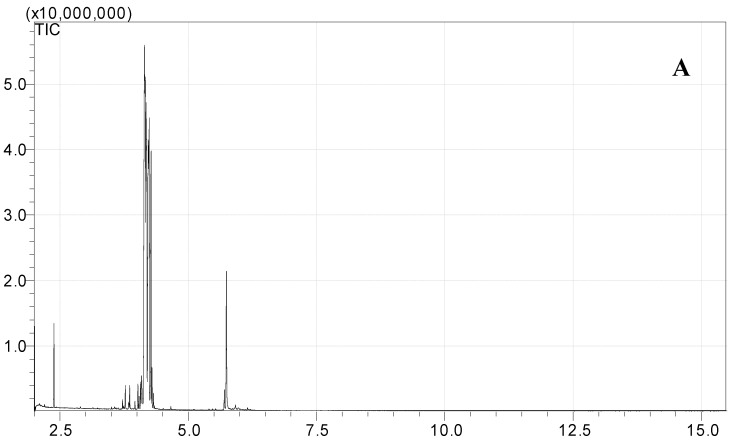

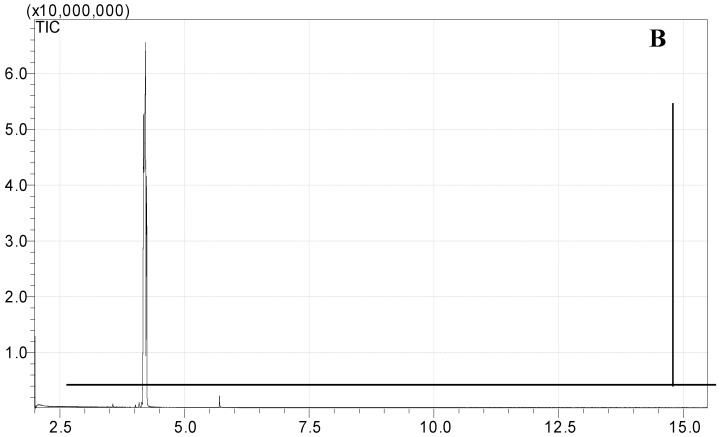
Total ion chromatogram (TIC) of standard mannose and date juice. For mannose, a peak was obtained at a retention time of 4.25 min. The spilt ratios were 40, instead of 70, in this analysis. (**A**) Chromatogram of date juice; (**B**) standard mannose chromatogram.

Figure 12 illustrates the chromatogram of standard mannose and date juice at a 40-split ratio. Standard mannose and date juice sugars were oximated and silylated. For mannose, a peak was obtained at a retention time of 4.25 min. Other than the spilt ratio, the operating conditions of the GCMS were the same (see Table 1). The spilt ratios were 40, instead of 70, in this analysis.

### 3.2. Quantification of the Sugar Concentration

The identified sugars were quantified using enzymatic assays. An enzymatic standard method and delta pH technique were used to determine the sugar concentration in the date juice. Two calibration curves were prepared to determination the sugar concentration in the date juice samples.

**Figure 13:**
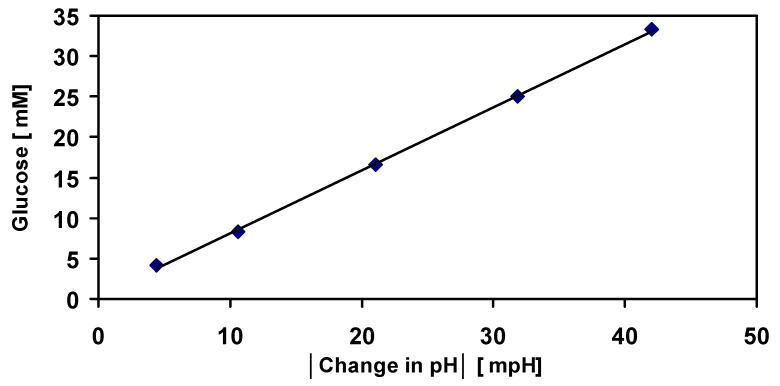
Calibration curve for the glucose concentration. Different glucose standards (4.4–42 mM) were analysed by measuring the change in the pH of the solution during the phosphorylation of glucose. This enzymatic reaction was achieved using a delta pH device. A calibration curve was obtained by plotting the glucose concentrations against the measured change in pH. The plotted values of the change in pH (mpH) are an average of three absolute measured values.

A method was developed to determine the sugar contents in date juice by measuring the change in the solution pH during glucose phosphorylation. A calibration curve was prepared by measuring the change in the solution pH against standard samples of different glucose concentrations, as shown in Figure 13. The plotted values for the change in pH are the average of three measured values with an average standard deviation of ±0.008%. A linear relationship was obtained between the change in pH and the glucose concentration (for glucose concentrations of 4.2–42 mM). The following equation revealed this linear relationship:

[Glucose] [mM] = [0.7798 × change in pH + 0.3508] × [mM](2)

Conducting residual analysis to the results demonstrates that the multiple R, the correlation coefficient, is 0.9997 (almost 1). Moreover, the coefficient of determination, the R squared, was 99.9%. This means, 99.9% of the variation of glucose concentration values around the mean are explained by the pH change -values. Additionally, the standard error of the regression was 0.35. These results can be found in Table 3. Therefore, the residual analysis of the results for the five observations confirmed the proposed linear relationship between the glucose concentration and the change in pH of the solution. In addition, Table 4 indicates the intercept and the slope of the line (e.g., 0.35 and 0.7798 respectively), which are shown in Equation (3).

**Figure 14:**
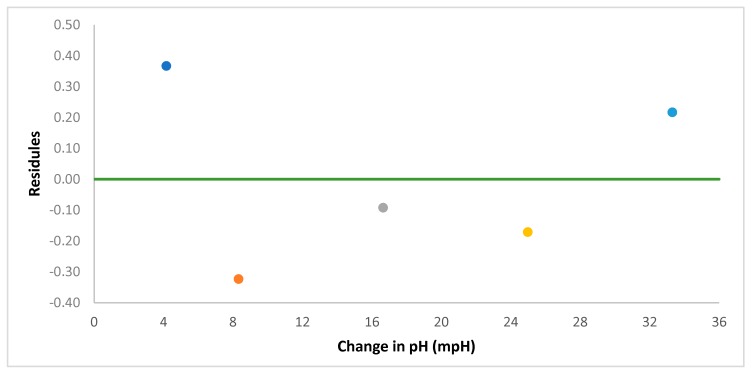
Residual plot of the data of glucose concentration that plotted against the measured change in pH.

The distribution of the points follows a random pattern as demonstrated in Figure 14. Furthermore, the residuals are relatively very small and that the sum up of these residuals is zero. Consequently, a linear model represents the overall pattern of the data.

**Figure 15:**
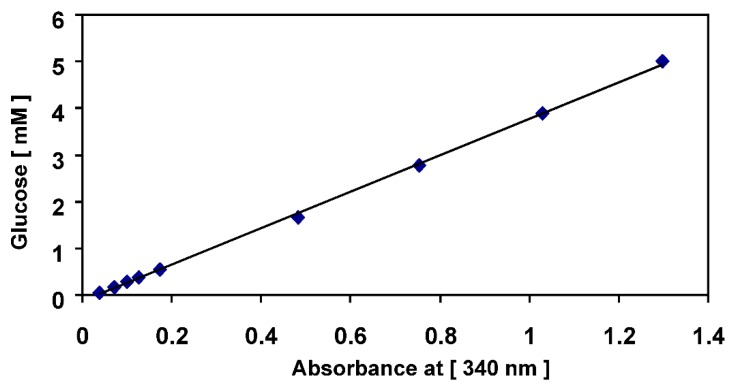
Calibration curve for the glucose concentration. Several glucose standards (0.05–5 mM) were analysed using an enzymatic kit assay (K-SUFRG). A calibration curve was obtained by plotting the glucose concentrations against their measured absorbance at 340 nm. The plotted values of absorbance are the average of three measured values with an average standard deviation of ±0.04.

Figure 15 represents a calibration curve of the absorbance values that were measured against different standard glucose concentrations. This calibration curve was obtained by following the enzymatic assay, K-SUFRG, described by Megazyme [15]. The plotted absorbance values are an average of three measured values with an average standard deviation of ±0.04. In this test, reasonable results were obtained for glucose concentrations (0.05–5 mM) because the calibration curve is a straight line in this concentration range. However, the sample must be diluted for glucose concentrations above 5 mM. The linear relationship between the glucose concentration and absorbance is outlined below:

[Glucose]
[mM] = [3.9015 × (Absorbance at 340 nm) − 0.1263] × [mM](3)

## 4. Discussion

### 4.1. Qualitative Analysis Using GCMS

Figure 1 shows the chromatograms of date juice. The retention times of the main peaks (e.g., 3.88, 3.905, 3.95, 3.90 and 5.33 min) were compared with the retention times of the individual standards of fructose (Figure 2), d-glucose (Figure 3) and sucrose (Figure 4). The retention times were identical; consequently, fructose, glucose and sucrose were detected in date juice. Furthermore, these results reveal two peaks for each of the six-carbon sugars, while only one peak was observed for sucrose; these results agree with the results of other reports [9,10]. Moreover, the mass spectra of the individual peaks of date juice were similar to the spectra of the analysed standard sugars. In addition, characteristic fragments (*m*/*z*) were obtained. A base peak was obtained at *m*/*z* 73. This fragment is characteristic of silylation and results from cleavage of the TMS group. Furthermore, fragments at (*m*/*z*) 103, 117, 147, 160, 191, 205, 217, 291 and 319 were observed for fructose and glucose, as shown in Figure 6, Figure 7, Figure 8 and Figure 9. These fragments are characteristic of glucose and fructose as demonstrated [22,23].

Additionally, a characteristic fragment for sucrose at (*m*/*z*) 361 is visible in each spectrum [23], as shown in Figure 10a,b. The results demonstrate that glucose and fructose are the main sugars in date juice and they are present in approximately equal amounts, while sucrose is a minor component. The identified sugars were detected using the enzymatic assays, as mentioned above. The GCMS results are in excellent agreement with the reported results and the results from the enzymatic methods.

Figure 1c shows a minor peak at 5.3 min. This peak could represent pyridine because a similar peak was observed for the blank pyridine sample, as shown in Figure 5. The GCMS database suggested that the spectrum was that of pyridine. In Figure 1a, a small peak was observed at 2.3 min. A similar peak was obtained during an earlier analysis of date juice at a split ratio of 40. At this spilt ratio, the standard mannose peak appeared at 4.25 min, as shown in Figure 12a,b. The GCMS library suggested that this peak is related to glycine in the date juice. Moreover, the company that produced the date juice reported that it includes 1.3% protein, which could be the source of this peak.

### 4.2. Quantification of the Sugar Concentration in Date Juice Enzymatic Kit Assay

When the glucose concentration increased from 0.01 g/L to 0.9 g/L, the obtained net absorbance at 340 nm increased from 0.039 to 1.299. The increment in absorbance was caused by the increase in the amount of formed NADPH from glucose phosphorylation. The formed NADPH absorbs at 340 nm. Therefore, the amount of formed NADPH is stoichiometric with respect to the amount of d-glucose. In consequence, there is a correlation between the amount of d-glucose and absorbance. A calibration curve of absorbance was plotted against d-glucose concentrations, as shown in Figure 15. A straight-line plot was obtained, which indicates that this curve can be used to determine the glucose concentration in the samples.

An enzymatic assay, K-SUFRG, was performed according to the protocol provided by Megazyme [15]; it indicated that the concentrations of glucose, fructose and sucrose in 1 g of date juice were 0.2737 g, 0.277 g and 0.064 g, respectively. Therefore, the total inverted sugar (glucose + fructose) content was 0.5507 g while the entire sugar content was 0.615 g in 1 g of date juice. The glucose concentration in the sample was calculated by comparing the obtained absorbance of the analysed sample with that of the calibration curve (Figure 15) using Equation (1). The fructose:glucose ratio was 0.99. This ratio was reported to be 1:1 [3] or 1.03:1 [4]. These comparisons indicate that the results are reliable.

An additional hydrolysis step was performed using β-fructosidase to convert date juice sucrose to d-glucose and D-fructose. The sucrose concentration was calculated from the difference between the total sugar concentration after hydrolysis and the initial concentrations of glucose and fructose.

### 4.3. Measuring the pH Change in the Solution

A CL10 deferential pH sensor was used to determine the sugar content in the date juice; it provided a slightly different result from that obtained by applying the K-SUFRG assay. Thus, 1 g of date juice contains a total of 0.5275 g of six-carbon sugars (glucose + fructose) while it contains 0.068 g of sucrose. Therefore, the total sugar content in 1 g of date juice is 0.600 g. These values were obtained from the prepared calibration curve (Figure 14). The change in the pH of the solution was caused by liberated H+ during the glucose phosphorylation reaction [14]. This enzymatic reaction, glucose phosphorylation, converted d-glucose to gluconate-6-phosphate, while ATP was converted to ADP.

Date juice contains glucose and fructose; therefore, it was impossible to measure the initial fructose concentration using the CL10 device. The hexokinase (HK) enzyme can simultaneously catalyse d-glucose and D-fructose, as shown below:

(4) $$D\text{-Glucose}+\text{ATP G-6-P}\overset{\mathrm{HK}}{⟶}+\mathrm{ADP}$$

(5) $$D\text{-Fructose}+\text{ATP F-6-P}\overset{\mathrm{HK}}{⟶}+\mathrm{ADP}$$

Consequently, the change in pH represented the total liberated H+ for both reactions. This amount can be correlated to the entire sugar content (glucose and fructose).

The slightly different results from the methods described above could be caused by the different applications of each device, for example, spectrophotometer versus differential pH sensor. Such applications require varying steps. Sample dilution, noise from the device and even small measurement errors could affect the measurements, that is, providing slightly different measurement values. Moreover, the obtained values of date juice sugars are in the range mentioned in Table 5. Additionally, a slightly higher total sugar content (0.63 g in 1 g of date juice) was determined by the company that produced the date juice. The cause of this small difference in the total sugar content cannot be determined because the analysis method used by the company is unknown.

Both applied methods agree with the results given in the literature and the results determined by the company that produced the date juice.

## 5. Conclusions

This study revealed the rapid identification of sugars in date juice using GCMS. The sugar contents of natural products are not known; therefore, these results demonstrate the powerful application of GCMS prior to an enzymatic method. Moreover, GCMS can be used to identify the sugars and determine the required enzymatic methods for sugar quantification in date juice. Additionally, measuring the change in pH can be performed to quantify the sugars content in food products.

## Figures and Tables

**Table 1:** GCMS operating conditions (instrument: Shimadzu GCMS-QP 2010).

Parameter	Gas Chromatography	Mass Spectrometry
Pressure	459 kPa	-
Ionisation energy	70 eV	-
Total flow rate	64.7 mL/min	-
Column flow	0.87 mL/min	-
Linear velocity	59.5 cm/s	-
Purge flow	3.0 mL/min	-
Split ratio	70.0	-
Carrier gas	Helium	-
Column oven temperature	80 °C	-
Ion source temperature	-	250 °C
Interface temperature	-	280 °C
Start time	-	2 min
End time	-	7.5 min
Full scan	-	45–750 amu

**Table 2:** Sample composition used in the enzymatic assays.

Pipette into Cuvettes	Blank (Glucose Test)	Sample (Glucose Test)	Blank (Fructose Test)	Sample (Fructose Test)	Blank (Sucrose Test)	Sample (Sucrose Test)
Bidest water (µL)	473	451.5	468	446.5	425	403.5
Sample (µL)	-	21.5	-	21.5	-	21.5
Solution 1 (µL)	21.5	21.5	21.5	21.5	21.5	21.5
Solution 2 (µL)	21.5	21.5	21.5	21.5	21.5	21.5
Suspension 3 (µL)	5	5	5	5	5	5
Suspension 4 (µL)	-	-	5	5	5	5
Solution 6 (µL)	-	-	-	-	43	43

**Table 3:** Regression Statistics.

Regression Statistics	
Multiple R	0.999715238
R Square	0.999430557
Adjusted R Square	−1.66666667
Standard Error	0.328476556
Observations	5

**Table 4:** Regression Statistics.

	Coefficients	Standard Error	t Stat	*p*-Value	Lower 95%	Upper 95%
Intercept	0.350821377	0.278089	1.261545	0.296283	−0.53418	1.235824
delta pH	0.779763723	0.010746	72.56242	5.77 × 10^−6^	0.745565	0.813963

**Table 5:** Date palm/syrup constituents (mg/100 g).

	This Research	Palm Date (Iraq) [2]	Date Juice (Iraq) [24]	Palm Date (UAE) [6]	Palm Date [25]	Date Syrup (Libya) [8]	Date Syrup (Egypt) [26]	Date Syrup [27]	Palm Date (Kuwait) [7]
Na	-	4.45	12	2.3–5.1	-	70.4	-	-	595–673
K	-	701	858	402–652	-	217	521	-	497.9–531.8
Ca	-	58	106.8	43–56	-	37.7	65	-	26–35.4
Mg	-	54.4	58.1	43.6–53.3	-	-	20	-	22.7–28.4
Fe	-	150	-	1.38–2.17	-	9.3	2.69	-	0.1
Cu	-	0.19	-	0.27–0.35	-	-	-	-	0.13–0.18
P	--	58.52	126	48.8–68.2	-	-	72	-	138.1–152.3
S	-	47.4	-	-	-	-	-	-	-
Cl	-	269	-	-	-	-	-	-	-
Thiamine	-	0.7	-		-	-	0.08	-	-
Riboflavin	-	0.03	-		-	-	0.05	-	-
Nicotinic acid B3	-	-	-	-	-	-	2.2	-	
Protein	-	-	1.3	2.0–2.5	-	1.2	3	-	2.03–2.6
Sugar	60–61.5	60–70	63.6	78–79.39	-	70.81	73	-	-
Reducing sugar	-	-	-	-	-	67.01–68.42	-	-	87.53–88.02
Mn	-	-	-	0.31–0.44	-		-	-	0.2–0.29
Glucose	27.37	-	-	38.47–40.4	32	33.32	-	26.9–34.5	38.02–38.45
Fructose	27.7	-	-	38.55–39.95	30	30.93	-	29.1–33.2	39.12–39.69
Sucrose	6.4–6.8	-	-	-	-	1.08–3.97	-	-	-
Fructose: Glucose	0.99:1	-	-	1:1	0.93:1	0.93:1	-	-	1.03:1
Fructose + Glucose	52.75–55.07								
